# Detection of enterovirus RNA in peripheral blood mononuclear cells correlates with the presence of the predisposing allele of the type 1 diabetes risk gene *IFIH1* and with disease stage

**DOI:** 10.1007/s00125-022-05753-y

**Published:** 2022-07-22

**Authors:** Amir-Babak Sioofy-Khojine, Sarah J. Richardson, Jonathan M. Locke, Sami Oikarinen, Noora Nurminen, Antti-Pekka Laine, Kate Downes, Johanna Lempainen, John A. Todd, Riitta Veijola, Jorma Ilonen, Mikael Knip, Noel G. Morgan, Heikki Hyöty, Mark Peakman, Martin Eichmann

**Affiliations:** 1 Department of Virology, Faculty of Medicine and Health Technology, Tampere University, Tampere, Finland; 2 Exeter Centre of Excellence for Diabetes Research (EXCEED), Institute of Biomedical and Clinical Science, University of Exeter Medical School, Exeter, UK; 3 Fimlab Laboratories, Pirkanmaa Hospital District, Tampere, Finland; 4 Immunogenetics Laboratory, Institute of Biomedicine, University of Turku, Turku, Finland; 5 JDRF/Wellcome Trust Diabetes and Inflammation Laboratory, Department of Medical Genetics, Cambridge Institute for Medical Research, University of Cambridge, Addenbrooke’s Hospital, Cambridge, UK; 6 Present Address: Cambridge University Hospitals Genomics Laboratory, Cambridge University Hospital NHS Foundation Trust, Cambridge Biomedical Campus, Cambridge, UK; 7 Department of Pediatrics, University of Turku and Turku University Hospital, Turku, Finland; 8 Clinical Microbiology, Turku University Hospital, Turku, Finland; 9 Present Address: JDRF/Wellcome Diabetes and Inflammation Laboratory, Wellcome Centre for Human Genetics, Nuffield Department of Medicine, National Institute for Health and Care Research/Oxford Biomedical Research Centre, University of Oxford, Oxford, UK; 10 Department for Children and Adolescents, Oulu University Hospital, Oulu, Finland; 11 Department of Paediatrics, Medical Research Center Oulu, University of Oulu, Oulu, Finland; 12 Pediatric Research Center, Children’s Hospital, University of Helsinki and Helsinki University Hospital, Helsinki, Finland; 13 Research Program for Clinical and Molecular Metabolism, Faculty of Medicine, University of Helsinki, Helsinki, Finland; 14 Center for Child Health Research, Tampere University Hospital, Tampere, Finland; 15 Department of Immunobiology, Faculty of Life Sciences & Medicine, King’s College London, London, UK; 16 National Institute for Health Research, Biomedical Research Centre at Guy’s and St Thomas’ National Health Service Foundation Trust, King’s College London, London, UK

**Keywords:** Autoimmunity, Enterovirus, Genetic risk, *IFIH-1*, Interferon induced with helicase C domain 1, MDA5, Melanoma differentiation-associated protein 5, Pancreatic islets, rs1990760, Type 1 diabetes

## Abstract

**Aims/hypothesis:**

Enteroviral infection has been implicated consistently as a key environmental factor correlating with the appearance of autoimmunity and/or the presence of overt type 1 diabetes, in which pancreatic insulin-producing beta cells are destroyed by an autoimmune response. Genetic predisposition through variation in the type 1 diabetes risk gene *IFIH1* (interferon induced with helicase C domain 1), which encodes the viral pattern-recognition receptor melanoma differentiation-associated protein 5 (MDA5), supports a potential link between enterovirus infection and type 1 diabetes.

**Methods:**

We used molecular techniques to detect enterovirus RNA in peripheral blood samples (in separated cellular compartments or plasma) from two cohorts comprising 79 children or 72 adults that include individuals with and without type 1 diabetes who had multiple autoantibodies. We also used immunohistochemistry to detect the enteroviral protein VP1 in the pancreatic islets of post-mortem donors (*n*=43) with type 1 diabetes.

**Results:**

We observed enhanced detection sensitivity when sampling the cellular compartment compared with the non-cellular compartment of peripheral blood (OR 21.69; 95% CI 3.64, 229.20; *p*<0.0001). In addition, we show that children with autoimmunity are more likely to test positive for enterovirus RNA than those without autoimmunity (OR 11.60; 95% CI 1.89, 126.90; *p*=0.0065). Furthermore, we found that individuals carrying the predisposing allele (946^Thr^) of the common variant in *IFIH1* (rs1990760, Thr946Ala) are more likely to test positive for enterovirus in peripheral blood (OR 3.07; 95% CI 1.02, 8.58; *p*=0.045). In contrast, using immunohistochemistry, there was no correlation between the common variant in *IFIH1* and detection of enteroviral VP1 protein in the pancreatic islets of donors with type 1 diabetes.

**Conclusions/interpretation:**

Our data indicate that, in peripheral blood, antigen-presenting cells are the predominant source of enterovirus infection, and that infection is correlated with disease stage and genetic predisposition, thereby supporting a role for enterovirus infection prior to disease onset.

**Graphical abstract:**

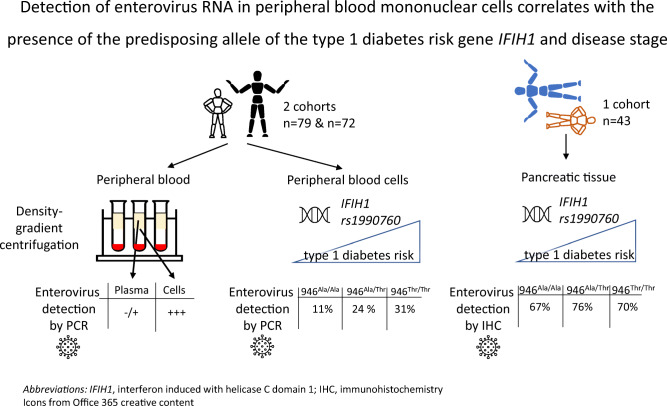

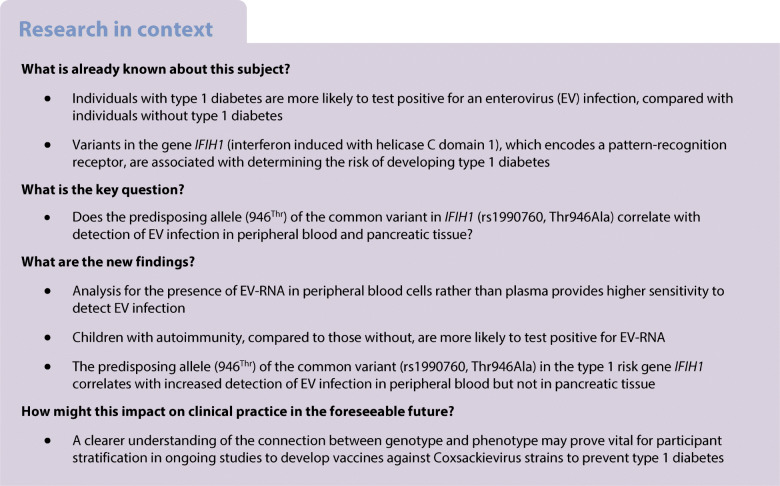



## Introduction

Type 1 diabetes is caused by progressive loss of the insulin-producing beta cells in pancreatic islets. Genetic factors are important in the predisposition to disease development [1]. However, a concordance rate of only around 50% in monozygotic twins [2] and the steadily increasing incidence rate [3], particularly in those individuals with lower genetic predisposition [3, 4], suggest that environmental factors also play a crucial role.

A prominent candidate environmental factor is virus infection [5], particularly infection with Coxsackievirus, a subgroup of the genus *Enterovirus* (EV) (*Picornaviridae* family) that has been extensively studied and linked to type 1 diabetes [6, 7]. EV is detectable at a higher frequency in stool samples [8, 9], pancreatic biopsies [10–12] and the peripheral blood [13, 14] of individuals with type 1 diabetes compared to those without, while the presence of neutralising antibodies against Coxsackievirus correlates with beta cell autoimmunity [15]. Studies have shown that EV is found more often in both the serum/plasma and peripheral blood mononuclear cells (PBMCs) [14, 16, 17] of individuals with type 1 diabetes and those with islet autoimmunity. Similarly, EV infection is detected in the pancreatic tissue of approximately 70% of post-mortem donors with recent-onset type 1 diabetes compared with less than 10% of similarly aged post-mortem donors without type 1 diabetes [10].

Mechanistically, there is an interaction between EV infection and the genetic variation that predisposes to type 1 diabetes. Several risk-determining variants have been identified in the gene *IFIH1* (interferon induced with helicase C domain 1), which encodes the cytoplasmic viral pattern-recognition receptor melanoma differentiation-associated protein 5 (MDA5) [18, 19]. MDA5 is essential for the detection of members of the *Picornaviridae* family [20, 21], and its activation leads to production of type I IFN and proinflammatory cytokines [22]. Most informatively, four rare SNPs exist that reduce or abrogate the function of MDA5, and these variants all provide protection against type 1 diabetes [19, 23]. For the common variant SNP rs1990760 (Thr946Ala) in *IFIH1*, 946^Thr^ is the predisposing allele [18]. In PBMCs, the disease-protective allele (946^Ala^) is associated with reduced expression of *IFIH1* either under basal conditions [24] or after stimulation with IFNβ or polyinosinic-polycytidylic acid [25, 26]. Functionally, however, a greater degree of divergence has been reported, with one study finding that protection correlates with reduced type I IFN response [26], while this was not seen in other studies [23, 27]. Another study observed reduced type III IFN responses, but not reduced type I IFN responses, in virus-infected pancreatic islets from donors homozygous for the predisposing allele in *IFIH1* [28].

Whether these functional consequences of variants in *IFIH1* affect the rate of virus infection and clearance is still under investigation, and the studies that have investigated the relationship between detection of EV and variants in *IFIH1* have yielded inconclusive results [8, 29]. Here we investigated whether detection of EV infection in peripheral blood and pancreatic tissue correlated with the predisposing allele (946^Thr^) of the common variant in *IFIH1* (rs1990760, Thr946Ala).

## Methods

### Cohorts

We analysed two distinct cohorts: the ‘children cohort’, which included 79 children (median age 119 months, range 17–192 months, 54% female); and the ‘adult cohort’, which included 72 adults (median age 29 years, range 18–51 years, 61% female). Ethics approval was obtained from the Bromley National Research Ethics Service Committee (reference number 08/H0805/14) for the adult cohort, and from the Ethics Committee of Pirkanmaa Hospital District, Tampere, Finland, for the children cohort. Written informed consent was obtained from all participants or their legal guardians.

The children cohort included 49 case children who repeatedly tested positive for multiple biochemical islet autoantibodies (referred to as mAAb-positive) (i.e. combinations of insulin autoantibodies (IAA), GAD autoantibodies (GADA) and tyrosine phosphatase IA-2 autoantibodies (IA-2A)) and 30 autoantibody-negative control children who were matched for age (all <13 years old), sex and place of birth (city). Among the children who were positive for mAAb, 24 later progressed to type 1 diabetes, diagnosed according to the WHO recommendations [30]. Both case and control children carried HLA genotypes that confer increased risk for type 1 diabetes, and had been followed from birth in the Finnish Type 1 Diabetes Prediction and Prevention study described previously [31]. PBMCs and plasma were isolated by density gradient centrifugation (Ficoll-Paque PLUS, GE Healthcare BioSciences, Sweden). PBMCs were pelleted and stored in RLT buffer (Qiagen, Germany). Both PBMCs and plasma were stored at -80°C for subsequent RNA extraction.

The adult cohort (all >18 years old) included 37 individuals with recent-onset type 1 diabetes (within 3 months of diagnosis) and 35 individuals without type 1 diabetes, of similar age and matched for sex, and with no family history of autoimmune disease. PBMCs were isolated by density gradient centrifugation (Lymphoprep; Axis-Shield, Norway). PBMCs were treated with FcR blocking reagent (Miltenyi Biotec, Germany), and PBMC subsets were subsequently enriched using magnetic bead cell separation by autoMACS (Mitenyi Biotec) in the following order: B cells (using CD19 MicroBeads), monocytes (using CD14 MicroBeads), myeloid dendritic cells (mDCs) (using a CD1c [BDCA-1] dendritic cell isolation kit), plasmacytoid dendritic cells (pDCs) (using a CD304 [BDCA-4/neuropilin-1] MicroBead kit). All reagents for cell separation were obtained from Miltenyi Biotec, and the post-separation enrichment was >90%, according to the manufacturer. Samples were pelleted and stored at -80°C until RNA extraction.

For both cohorts, individuals who reported or showed symptoms of systemic ‘virus-like’ illness were not recruited to the study or did not undergo blood sampling. In the children cohort, none of the individuals were excluded from blood sampling due to ‘virus-like’ illness.

### RNA extraction and detection of EV-RNA

RNA was extracted using a QIAamp viral RNA kit (Qiagen) and TRIzol reagent (Life Technologies, USA), in the adult and children cohorts, respectively, according to the manufacturer’s instructions. Detection of EV-RNA was performed by RT-PCR and liquid-phase hybridisation using a primer pair (forward: 5′-CGGCCCCTGAATGCGGCTAA-3′; reverse: 5′-GAAACACGGACACCCAAAGTA-3′) from the highly conserved 5′ non-coding region as previously described [32]. PCR amplicons were hybridised using a europium-labelled EV-specific probe (5′-TAITCGGTTCCGCTGC-3′) in a liquid-phase assay on a microtitre plate [33]. All positive samples were confirmed as positive by repeated RT-PCR and hybridisation assay.

### *IFIH1* genotyping

In the adult cohort, DNA was extracted from whole blood collected using the QIamp blood mini kit (Qiagen) according to the manufacturer’s instructions, and genotyping for the SNP rs1990760 was performed by TaqMan assay (Applied Biosystems, USA). In the children cohort, DNA was extracted from EDTA-treated blood samples by a salting-out protocol [34], and genotyping was performed either using a Sequenom platform (San Diego, USA) at the Genome Center of Eastern Finland, University of Eastern Finland (Kuopio), or by TaqMan assay (Applied Biosystems) in samples that were not included in the previous Sequenom-based study [35]. For each pancreas, sample DNA was extracted from 2 × 4 μm formalin-fixed, paraffin-embedded (FFPE) tissue curls using the QIAamp DNA FFPE tissue kit (Qiagen) according to the manufacturer’s instructions. SNP genotyping was performed by Kompetitive allele-specific PCR (KASP) (LGC Biosearch Technologies, UK) using 1 μl DNA amplified in a 5 μl KASP reaction. DNA was amplified and fluorescence detected using the QuantStudio 12K Flex Real-Time PCR system (ThermoFisher). Genotypes were called using QuantStudio 12K Flex software version 1.2.2 (ThermoFisher). We were unable to isolate pure and good-quality DNA from formalin-fixed, paraffin-embedded tissue for all donors, and therefore obtained *IFIH1* genotypes for 43 of the previously reported 72 post-mortem donors with type 1 diabetes [10].

### Immunohistochemistry

Formalin-fixed, paraffin-embedded pancreatic tissue from 43 individuals (median age 13.5 years, range 1–42 years, 69% female) with recent-onset type 1 diabetes, whose pancreatic histology has been described previously [36], was used for the immunohistochemical study. Data for the staining of the enteroviral protein VP1, and representative staining images, have been reported previously [10]. As previously described, VP1 positivity was assigned when at least one intensely stained endocrine cell was present in any islet within any given section [10]. All samples were used with ethical permission from the West of Scotland Research Ethics Committee (reference 20/WS/0074; Integrated Research Application System project ID 28362015/WS/0258). Sections were processed and labelled using a standard immunoperoxidase technique for paraffin sections, using heat-induced epitope retrieval. Sections to be labelled with Dako anti-vp1 (5D8/1; Dako Cytomation, UK) were heated in 1 mmol/l EDTA, pH 8.0. Primary antibodies were applied for 30 min at room temperature, and a Dako REAL EnVision detection system was used for antigen detection [10].

### Statistical analysis

Sample size calculation with a power of 0.8 predicted that a sample size of 69 was required to detect a threefold increase in EV detection sensitivity from a proportion in population 1 (p1)=0.1 to p2=0.3. Statistical analysis was performed using GraphPad Prism (version 8, GraphPad Software, USA). Odds ratios and *p* values were calculated using two-sided Fisher’s exact test, and 95% confidence intervals were computed using the Baptista–Pike method [37]. A *p* value <0.05 was considered statistically significant. Power analysis (post hoc and a priori) was performed using G*Power (version 3.1.9.7) [38].

## Results

### Enhanced detection of EV-RNA in the cellular compartment of peripheral blood

**Fig. 1:**
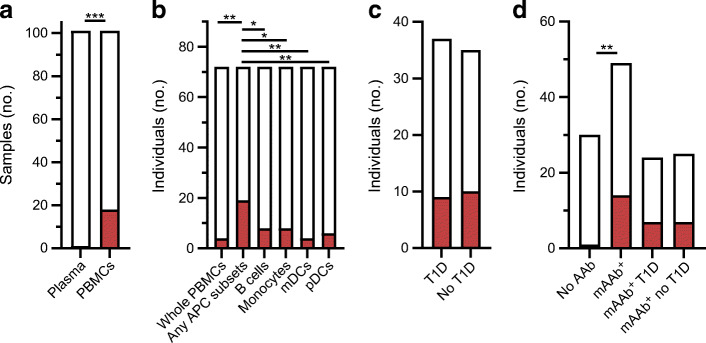
Detection of EV-RNA in peripheral blood. In the children cohort, the presence of EV-RNA was assessed in plasma and PBMCs (**a**) and in PBMCs from specific subgroups (**d**). In the adult cohort, the presence of EV-RNA was assessed in defined peripheral blood cell subsets (**b**) and in individuals with and without type 1 diabetes (**c**). Red shading indicates EV-RNA-positive; white indicates EV-RNA-negative. Differences between groups were statistically significant as indicated: *p<0.05; **p<0.01; ***p<0.001 (Fisher’s exact test, two-sided). T1D, type 1 diabetes

The presence of EV-RNA was evaluated in various peripheral blood fractions in individuals with type 1 diabetes, mAAb-positive individuals and individuals with neither type 1 diabetes nor autoantibody. We first aimed to establish which compartment in peripheral blood provides the highest sensitivity for detection of EV-RNA. We tested plasma and PBMCs isolated by density gradient centrifugation from the same blood drawn on 101 occasions from a total of 79 children in our children cohort. We found superior sensitivity to detect EV-RNA in the cellular compartment (i.e. PBMCs), in which 18 of 101 samples (17.8%) tested positive for EV-RNA, compared with the non-cellular compartment (i.e. plasma) in which 1 of 101 samples (1.0%) tested positive for EV-RNA (OR 21.69; 95% CI 3.64, 229.20; *p*<0.0001) (Fig. 1a). In the one instance where positivity was seen in the plasma sample, the PBMC sample also tested positive for EV-RNA. To further pinpoint the cellular compartment that harbours EV-RNA, we tested four immune cell subsets in addition to whole PBMCs for the presence of EV-RNA in a cohort of adults with and without type 1 diabetes. These subsets were B cells, monocytes, mDCs and pDCs, all representing antigen-presenting cells (APCs).

We detected EV-RNA in a higher proportion of individuals when analysing APC subsets combined (26.4%, 19/72) than when analysing whole PBMCs from the same individuals (5.6%, 4/72) (OR 6.09; 95% CI 2.10, 17,17; p=0.0011) (Fig. 1b). Individuals who tested positive for EV-RNA in whole PBMCs also tested positive for EV-RNA in at least one subset of APCs. Two of these individuals tested positive in the monocyte subset, one in the B cell subset, and one in all APC subsets. Among all individuals who tested positive for EV-RNA, EV-RNA was detected in the B cell subset for eight individuals, in the monocyte subset for eight individuals, in the mDC subset for four individuals, in the pDC subset for six individuals, and in whole PBMCs for four individuals. We did not find a difference in the sensitivity for detection of EV-RNA between the different subsets of APCs. Overall, we detected EV-RNA in the cellular compartment (i.e. PBMCs) of 15/79 individuals in the children cohort (19.0%) and 19/72 individuals in the adult cohort (26.4%) (Fig. 1d and b, respectively).

### EV-RNA detection correlates with autoimmunity and disease in children but not in adults

Next we investigated whether positivity for EV-RNA in PBMCs correlates with defined stages of type 1 diabetes, i.e. adults with recently diagnosed type 1 diabetes (<3 months) and children positive for mAAb with an ongoing autoimmune reaction.

In the adult cohort, we detected EV-RNA in nine of 37 individuals with type 1 diabetes (24.3%) compared with 10 of 35 individuals without type 1 diabetes (28.6%) (*p*=0.79) (Fig. 1c). In the children cohort, EV-RNA was detected in PBMCs in 14 of 49 children with mAAb (with or without type 1 diabetes) (28.6%) compared with one of 30 matched control children (without autoantibody or type 1 diabetes) (3.3%) (OR 11.60; 95% CI 1.89, 126.90; *p*=0.0065) (Fig. 1d).

### Increased detection of EV-RNA in peripheral blood but not tissue in individuals carrying the common type 1 diabetes-predisposing allele in *IFIH1*

We next investigated whether detection of EV infection (by detecting EV-RNA or VP1) in individuals correlates with the predisposing allele (946^Thr^) of the common variant in *IFIH1* (rs1990760, Thr946Ala). The distribution of the common variant in *IFIH1* in cohorts, and detection of EV-RNA according to subgroup and genotype, is summarised in Table 1. We found that homozygosity for the protective allele (946^Ala^) significantly reduced the OR to detect EV-RNA in both the recessive model (homozygous protective vs homozygous risk: OR 0.26; 95% CI 0.087, 0.84; reciprocal of OR 3.81; 95% CI 1.19, 11.46; *p*=0.031) and the additive protective model (homozygous protective vs homozygous risk and heterozygous: OR 0.33; 95% CI 0.12, 0.98; reciprocal of OR 3.07; 95% CI 1.02, 8.58; *p*=0.045), when analysing the children and adult cohorts in combination (Table 2). In the adult and children cohorts, respectively, EV-RNA was detected in 34.6% (9/26) and 25.0% (4/16) of individuals who were homozygous for the predisposing allele, 26.5% (9/34) and 21.6% (8/37) of individuals who were heterozygous, and 8.3% (1/12) and 11.5% (3/26) of individuals who were homozygous for the protective allele of the common variant in *IFIH1* (Table 1).

**Table 1:** Detection of EV-RNA and *IFIH1* genotype in the children and adult cohorts

Cohorts and subgroups	*IFIH1* variants			Total
	946^Ala/Ala^	946^Ala/Thr^	946^Thr/Thr^	EV-RNA^pos^/ total
Children				
No islet autoantibody	0/9 (0.0)	0/13 (0.0)	1/8 (12.5)	1/30 (3.3)
mAAb				
With T1D	1/9 (11.1)	4/12 (33.3)	2/3 (66.7)	7/24 (29.2)
Without T1D	2/8 (25.0)	4/12 (33.3)	1/5 (20.0)	7/25 (28.0)
Sub-total	3/17 (17.7)	8/24 (33.3)	3/8 (37.5)	14/49 (28.6)
All children	3/26 (11.5)	8/37 (21.6)	4/16 (25.0)	15/79 (19.0)
Adult				
With T1D	0/5 (0.0)	3/17 (17.7)	6/15 (40.0)	9/37 (24.3)
Without T1D	1/7 (14.3)	6/17 (35.3)	3/11 (27.3)	10/35 (28.6)
All adults	1/12 (8.3)	9/34 (26.5)	9/26 (34.6)	19/72 (26.4)
Children and adult combined	4/38 (10.5)	17/71(23.9)	13/42 (31.0)	34/151 (22.5)

Data are shown as EV-RNA-positive individuals/total individuals (frequency of EV-RNA positivity expressed as %)

T1D, type 1 diabetes

**Table 2:** OR for detection of EV-RNA according to *IFIH1* variants

	Combined		Adult		Children	
*IFIH1* 946 variant	OR (95% CI)	*p* value	OR (95% CI)	*p* value	OR (95% CI)	*p* value
Ala/Ala vs Thr/Thr	0.26 (0.087, 0.84)	0.031*	0.17 (0.015, 1.22)	0.13	0.39 (0.089, 1.69)	0.40
Ala/Ala vs Ala/Thr	0.37 (0.13, 1.18)	0.13	0.25 (0.021, 1.71)	0.25	0.47 (0.13, 1.91)	0.50
Ala/Ala + Ala/Thr vs Thr/Thr	0.53 (0.23, 1.21)	0.13	0.53 (0.18, 1.56)	0.27	0.64 (0.19, 2.08)	0.49
Ala/Ala vs Ala/Thr + Thr/Thr	0.33 (0.12, 0.98)	0.045*	0.21 (0.019, 1.47)	0.16	0.45 (0.13, 1.73)	0.36

OR were calculated using the Baptista–Pike method

*p* values are for EV-RNA-positive vs EV-RNA-negative (Fisher’s exact test, two-sided); *p<0.05

**Fig. 2:**
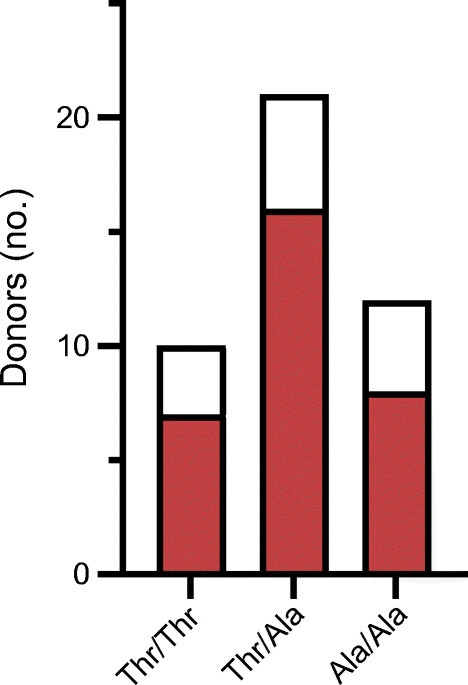
Detection of EV capsid protein VP1 in pancreatic islet sections. EV capsid protein VP1 was detected by immunohistochemistry in tissues from 43 donors with type 1 diabetes, with the defined variant in *IFIH1* (rs1990760, Thr946Ala). Red shading indicates EV-RNA-positive; white indicates EV-RNA-negative. Differences between groups were not statistically significant (Fisher’s exact test, two-sided)

We then explored whether the correlation between the protective allele (946^Ala^) and reduced detection of EV infection in the cellular compartment of peripheral blood also extends to pancreatic islets studied in situ. To this end, we assessed the presence of the EV capsid subunit viral protein 1 (VP1) in pancreatic tissue sections recovered from 43 donors with type 1 diabetes and held within the Exeter Archival Diabetes Biobank (data reported previously by Richardson et al [10]). VP1 was detected in the pancreatic islets of 72.1% of the donors (31/43). Detection of VP1 did not correlate with predisposing allele (946^Thr^) of the common variant (rs1990760, Thr946Ala) in *IFIH1*. VP1 was detected in the pancreatic islets of 70.0% (7/10), 76.2% (16/21) and 66.7% (8/12) of donors with the homozygous risk variant, those who were heterozygous, and those with the homozygous protective common variant (rs1990760, Thr946Ala) in *IFIH1*, respectively (Fig. 2).

## Discussion

Our data from the children cohort show a significantly increased sensitivity for detection of EV-RNA within the cellular compartment of peripheral blood compared with plasma. Additionally, using the adult cohort, we found that EV infection was detected in more individuals when APC subsets (B cells, monocytes, mDCs and pDCs) were analysed for EV-RNA, compared with whole PBMCs. These observations had statistical power (post hoc) of >0.9. Hence, our data indicate that APCs are ‘carriers’ of EV-RNA in peripheral blood as every individual that tested positive for EV-RNA in the PBMC sample also tested positive for EV-RNA in at least one subset of APCs. Similar observations, that EV-RNA is found more frequently in PBMCs than serum, have been made previously, albeit in a smaller cohort [14]. We postulate that APCs are carriers of EV-RNA because they pick up enterovirus in infected tissues or because these cells are sites of active viral replication, as suggested previously [39, 40]. EV infection in APCs may markedly modulate their function and efficacy of viral and autoantigen presentation. Infected APCs may also serve as a carrier to transport virus to uninfected tissues.

Our analysis shows that positivity for EV-RNA is associated with islet autoimmunity. Children positive for mAAb were more likely to test positive for EV-RNA than those without mAAb (post hoc statistical power 0.85). In children positive for mAAb, we found a similar frequency of EV-RNA positivity among children who later progressed to type 1 diabetes and those who have not yet progressed. In the adult cohort, we did not detect a correlation (*p*=0.79) between positivity for EV-RNA and type 1 diabetes, potentially due to the increased sensitivity of detection of EV-RNA in PBMC subsets. Overall, our findings are in line with the results of previous studies summarised in meta-analyses by Yeung et al and Wang et al [6, 7], the majority of which reported increased detection of EV infection in individuals with autoimmunity and/or type 1 diabetes compared to those without.

Given the ‘snapshot’ nature of this and previous studies [14, 41] and the fact that EV viraemia lasts for only up to two weeks in peripheral blood [13], we suggest that larger study cohorts, longitudinal sampling, and improved sensitivity of viral detection (as shown here) are likely to be needed to reveal significant differences. This may be achieved using cohort studies such as the Finnish Type 1 Diabetes Prediction and Prevention study, which regularly sample children longitudinally. It is also probable that genetic variation, rather than disease stage, defines the effectiveness of the antiviral response, the rate of viral clearance and the level and spread of any EV infection, and therefore influences the detection of EV-RNA.

To obtain a larger cohort, we combined our two cohorts, and found that individuals carrying the predisposing allele (946^Thr^) of the common variant in *IFIH1* (rs1990760, Thr946Ala) were more likely to test positive for EV-RNA than those without the predisposing allele (in both the additive and protective recessive models). However, our results are based on a limited sample size and low statistical power (post hoc) (0.62 and 0.54 for the recessive and additive protective models, respectively). In the few studies reported so far, no correlation was found between *IFIH1* (rs1990760, Thr946Ala) homozygous genotypes and EV-RNA detection in peripheral blood [14, 29] or faecal samples [8]. Our results, and the proposed methodology for improved EV-RNA detection, suggests that further studies, with an increased sample size (power of 0.8 predicted at *n*=68 per homozygous group, based on our reported proportions of EV-RNA detection per group) should allow definition of the relationship between the *IFIH1* Thr946Ala genotype and EV infection detected in peripheral blood.

A potential limitation of our study is that symptoms observed in individuals with recent-onset type 1 diabetes (particularly children) may overlap with those of a virus infection. This overlap in symptoms may introduce a sampling bias between study groups (i.e. individuals without type 1 diabetes and individuals with recent-onset type 1 diabetes) if symptoms observed in individuals with recent-onset type 1 diabetes are misinterpreted as the exclusion criterion, or vice versa. However, we did not observe a sampling bias with regard to the exclusion criterion ‘virus-like’ illness in the children cohort. In the adult cohort, none of the sampled participants exhibited symptoms of ‘virus-like’ illness at the time of recruitment and sampling. However, we do not have data on individuals that were not recruited to the study due to meeting the exclusion criterion. Therefore, we cannot state whether such a sampling bias occurred in the adult cohort. Thus, while we think it unlikely that a sampling bias occurred between the study groups of individuals without type 1 diabetes and with recent-onset type 1 diabetes in the adult cohort, we cannot exclude this.

We then further tested whether our finding of an association between the predisposing allele of the common variant and increased EV-RNA detection in peripheral blood extends to pancreatic tissue of post-mortem donors with type 1 diabetes. As previously reported, we detected the EV capsid protein VP1 in pancreatic islets in the majority (>70%) of donors with type 1 diabetes [10, 12]. Here we report that we did not detect a correlation between the predisposing allele (946^Thr^) of the common variant in *IFIH1* (rs1990760, Thr946Ala) and the presence of EV infection (i.e. positivity for VP1) in pancreatic islets. This probably reflects the fact that most individuals with type 1 diabetes display signs of EV infection in pancreatic islets, and that the effect of the variant in *IFIH1* may be more nuanced than simply the presence or absence of VP1 positivity in islets.

Our finding of a significantly increased prevalence of EV-RNA in children positive for mAAb, regardless of their *IFIH1* genotype, suggests that a dysregulated immune response and ongoing autoimmunity may interfere with the control and/or clearance of EV infection. We cannot exclude the possibility that infection of pancreatic islet cells by enterovirus is influenced by genetic predisposition. Our analysis focused solely on detection of immunopositivity for the capsid protein VP1. Future analysis of the level of expression of VP1 within islet cells and/or the frequency of VP1-positive cells within pancreatic islets may provide further insights into the effects of genetic predisposition to type 1 diabetes by the common variant in *IFIH1* (rs1990760, Thr946Ala).

In summary, our results indicate a correlation between the ability to detect EV-RNA in the cellular compartment of peripheral blood and the presence of the predisposing allele (946^Thr^) of the common variant (rs1990760, Thr946Ala) in the type 1 diabetes risk gene *IFIH1*. We also show that enterovirus is detected more often in children with islet autoimmunity compared to those without. Our data further support the view that analysis of APCs increases the sensitivity for detection of EV infection in peripheral blood, and that EV infection is part of the aetiology of type 1 diabetes. Ongoing studies for development of vaccines against Coxsackievirus strains to prevent type 1 diabetes will also be informative [42], and may require consideration of genotype/phenotype information for stratification of participants in trials.

## Data Availability

The datasets generated during and/or analysed during the current study are available from the corresponding author on reasonable request.

## Abbreviations

APC: Antigen-presenting cell
EV: Enterovirus
mAAb: Multiple autoantibodie(s)
MDA5: Melanoma differentiation-associated protein 5
mDC: Myeloid dendritic cell
PBMC: Peripheral blood mononuclear cell
pDC: Plasmacytoid dendritic cell
VP1: Viral protein 1
